# CycleGAN models show consistent brain MRI synthesis across datasets supporting downstream tissue characterization in multiple sclerosis

**DOI:** 10.3389/fninf.2026.1762794

**Published:** 2026-03-12

**Authors:** Shayan Shahrokhi, Olayinka Oladosu, Rehman Tariq, Yunyan Zhang

**Affiliations:** 1 Neuroscience Graduate Program, Faculty of Graduate Studies, University of Calgary, Calgary, AB, Canada; 2 Hotchkiss Brain Institute, Cumming School of Medicine, University of Calgary, Calgary, AB, Canada; 3 Department of Radiology, University of Calgary, Calgary, AB, Canada; 4 Biomedical Engineering Graduate Program, Faculty of Graduate Studies, University of Calgary, Calgary, AB, Canada; 5 Department of Clinical Neurosciences, University of Calgary, Calgary, AB, Canada

**Keywords:** brain MRI, CycleGAN, image synthesis, multiple sclerosis, Pix2Pix, spectral normalization

## Abstract

**Background:**

Secondary quantitative analysis of brain magnetic resonance imaging (MRI) can provide valuable information for many neurological diseases, including multiple sclerosis (MS), but it demands complete datasets that are often unavailable clinically. We investigated how image synthesis via deep learning using cycle-consistent generative adversarial networks (CycleGANs) compared with Pix2Pix as a related method, based on T1-weighted and T2-weighted brain MRI in MS, following verification on two streamlined datasets. The synthesized images were also evaluated against the source data.

**Methods:**

The streamlined datasets involved 1,113 healthy participants from the Human Connectome Project (HCP) and 318 participants from the Parkinson’s Progression Markers Initiative (PPMI). The MS cohort in this study included 105 participants scanned with different protocols. Image synthesis was bidirectional between T1- and T2-weighted MRI using CycleGAN with and without spectral normalization, as well as Pix2Pix. Utility testing focused on T1-weighted MRI that was most often unavailable in MS, and that involved lesion detection, brain volumetry, and lesion texture analysis.

**Results:**

All CycleGAN models performed competitively, while Pix2Pix performed better, mostly with streamlined datasets (*p* < 0.001). The average peak signal-to-noise ratio ranged from 24.860–28.570 versus 28.520–31.100, and the structural similarity index ranged from 0.838–0.901 versus 0.924–0.943. With spectral normalization, CycleGAN improved in PPMI but not in HCP and generally not in MS (*p* < 0.001). Furthermore, the synthesized images showed high similarity to the source data in utility tests, although Pix2Pix T1 images appeared more heterogeneous in lesion texture than source T1 images.

**Conclusion:**

CycleGAN without spectral normalization appeared feasible for synthesizing common clinical brain MRI, including T1-weighted images usable for subsequent quantitative analysis in MS.

## 1 Introduction

Magnetic resonance imaging (MRI) is an essential tool for the evaluation and management of neurological diseases such as multiple sclerosis (MS) (Thompson et al., 2018). Clinical MS imaging, including T1-weighted (T1w) and T2-weighted (T2w) brain MRI, provides valuable diagnostic information, such as the number and activity of focal lesions (McGinley et al., 2021). However, these features reflect only part of MS pathology (Fisniku et al., 2008). Secondary analysis of clinical MRI using computational approaches has the potential to characterize both visible and invisible tissue changes, thereby generating competitive data-driven hypotheses (Vázquez-Marrufo et al., 2023). Nonetheless, such approaches demand complete datasets, which are often limited in a clinical setting due to constraints in time or cost. There is a clear need to investigate new methods, such as deep learning techniques, that can synthesize missing imaging sequences from available data.

One common deep learning method for image synthesis is the generative adversarial network (GAN) (Goodfellow et al., 2020; Xu et al., 2022). GANs work by combining two neural networks, namely a generator and a discriminator, which create new images and determine their validity, respectively. These methods have shown promise in different applications, including synthesizing brain MRI to support segmentation of brain tumors (Nema et al., 2020) and MS lesions (Zhang et al., 2018). However, base models of GANs are not without limitations, as demonstrated by training instability in their discriminators due to inefficient learning, especially in cross-domain image translation (Lee and Choi, 2020). Developments are underway to overcome these challenges, including applying weight clipping (Arjovsky et al., 2017) or spectral normalization (weight control) (Lin et al., 2021) to the discriminators, and developing new architectures such as conditional GAN.

Two recognized conditional GANs are Pix2Pix and cycle-consistent GAN (CycleGAN). Pix2Pix targets paired image synthesis and is trained unidirectionally (Isola et al., 2017). CycleGAN, on the other hand, can handle unpaired images, enabled by the inclusion of two pairs of generators and discriminators (Zhu et al., 2017). Various studies demonstrate the utility of these models. With brain MRI of MS, Pix2Pix has assisted in image sequence translation and enhancement for cortical (Finck et al., 2022) and white matter lesion segmentation (La Rosa et al., 2021). CycleGAN has demonstrated competence in creating pseudo-healthy and lesion images (Basaran et al., 2022), and in adapting MRI between centers for segmenting white matter MS lesions (Julián Alberto et al., 2020). Furthermore, spectral normalization has demonstrated value in stabilizing CycleGAN in cross-modality image translation from computed tomography (CT) to MRI (Xu et al., 2020). However, current studies focus mainly on synthesizing images for tissue segmentation rather than structural analysis. Additionally, few studies have investigated synthesizing frequently non-acquired sequences, such as T1w brain MRI in MS, using routine clinical care data. Routine MRI is typically more heterogeneous than streamlined datasets in both scanner type and acquisition parameters (Eche et al., 2021), requiring extra attention.

The purposes of this study were threefold. The first was to investigate how CycleGAN models trained using large, streamlined datasets compared with our heterogeneous clinical MS data in image synthesis between T1w and T2w MRI. The second was to evaluate whether and how spectral normalization impacted CycleGAN performance. The third was to explore the utility of synthesized images versus source images in different analysis tasks, focusing on T1w MRI in MS. In contemporary MS protocols, T1w MRI is not always acquired in routine diagnostic or follow-up examinations (Wattjes et al., 2021); however, many image processing pipelines require a T1w sequence, as seen in co-registration and brain volume measurement (Jenkinson et al., 2012; Fischl, 2012). T2w brain MRI is commonly available in MS protocols (Wattjes et al., 2021), but the acquisition parameters often vary across scanners. Throughout the process, a Pix2Pix model was studied for comparison.

## 2 Methods

### 2.1 Data characteristics

Two publicly available datasets were included, with each acquired using streamlined protocols. One involved healthy brain MRIs from 1,113 participants (aged 22–36 years, 606 women) in the Human Connectome Project (HCP) (Van Essen et al., 2012). The other comprised brain MRIs from 318 participants (aged 60–70 years, 125 women) in the Parkinson’s Progression Markers Initiative (PPMI) (Marek et al., 2011). Data utilization followed all required practices.

Our local dataset involved clinical brain MRI from a sample of 169 participants with relapsing–remitting MS, who were enrolled in an ongoing cohort study known as Clinical Impact of Multiple Sclerosis (Ethics ID: REB14-1926). Of these, 104 participants (73 women) who had both T1w and T2w sequences available were selected. The mean ± standard deviation age was 36.38 ± 8.89 years, disease duration was 4.81 ± 6.88 years, and the median Expanded Disability Status Scale (EDSS) was 2.0. This study was approved by the Institutional Ethics Review Board, with written informed consent obtained from each participant.

### 2.2 Imaging protocol

The HCP scans were acquired with a 3T scanner (Magnetom Skyra, Siemens Healthineers, Erlangen, Germany). T1w imaging used a magnetization-prepared rapid gradient-echo (MPRAGE) sequence with a repetition/echo time (TR/TE) of 2,400 ms/2.14 ms, slice thickness of 0.7 mm, field of view (FOV) of 224 mm, and a matrix size of 256 × 320. T2w MRI used the Sampling Perfection with Application optimized Contrasts using different flip-angle evolutions (SPACE) sequence with TR/TE of 3,200 ms/565 ms, slice thickness of 0.7 mm, FOV of 224 mm, and the same matrix size.

The PPMI images were acquired using 1.5T and 3T Siemens scanners, where only baseline scans were used. T1w MRI used an MPRAGE sequence with a TR/TE of 3,000 ms/2.98 ms, slice thickness of 1 mm, FOV of 256 mm, and a matrix size of 240 × 256. T2w MRI used a turbo spin-echo sequence with a TR/TE of 3,000 ms/101 ms, slice thickness of 3 mm, FOV of 256 mm, and a matrix size of 240 × 256.

**Figure 1:**
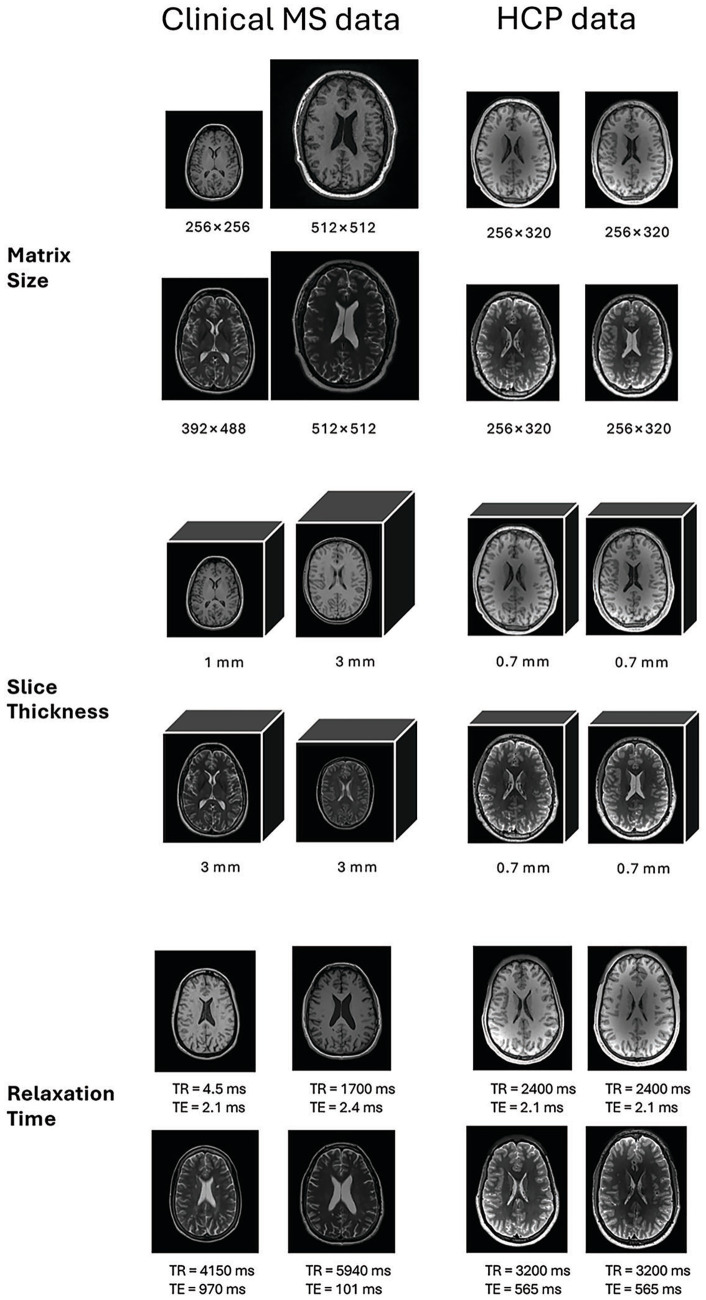
Example brain MRI protocols of high and low heterogeneity from clinically standard MS (left 2 columns) and streamlined HCP datasets (right 2 columns), respectively. Shown are T1-weighted (top rows) and T2-weighted (bottom rows) images associated with three common protocol settings: matrix size, slice thickness, and repetition and echo times (TR & TE) that can impact image size, resolution, and anatomical contrast. Under relaxation time, the T2-weighted MRIs of clinical MS also show different matrix sizes of 336 × 384 and 392 × 488, while the HCP images have the same image size.

Our MS imaging involved both Siemens and GE scanners at 1.5T or 3T. T1w MRI used either an MPRAGE or fast spoiled gradient-echo sequence, with TR of 4.3–2,490 ms, TE of 2.08–33.96 ms, slice thickness of 1–3 mm, and matrix size of 192 × 256 to 512 × 512. T2w MRI applied mainly a spin-echo sequence, with TR of 2,970–11,470 ms, TE of 83–122.90 ms, slice thickness of 3 mm, and matrix size of 256 × 256 to 512 × 512 (Figure 1).

### 2.3 Image preprocessing

Four steps were performed to optimize image quality. These included nonuniformity correction using the N4ITK algorithm of Advanced Normalization Tools (ANTs) (Tustison et al., 2010) and brain extraction using FSL (FMRIB Software Library, Oxford, UK). All scans were then linearly co-registered to the standard MNI152 1-mm T1 template using ANTs. Finally, image intensity was normalized to the range 0–1 for subsequent modeling.

### 2.4 Image synthesis using CycleGAN

#### 2.4.1 Model development for Vanilla CycleGAN

**Figure 2:**
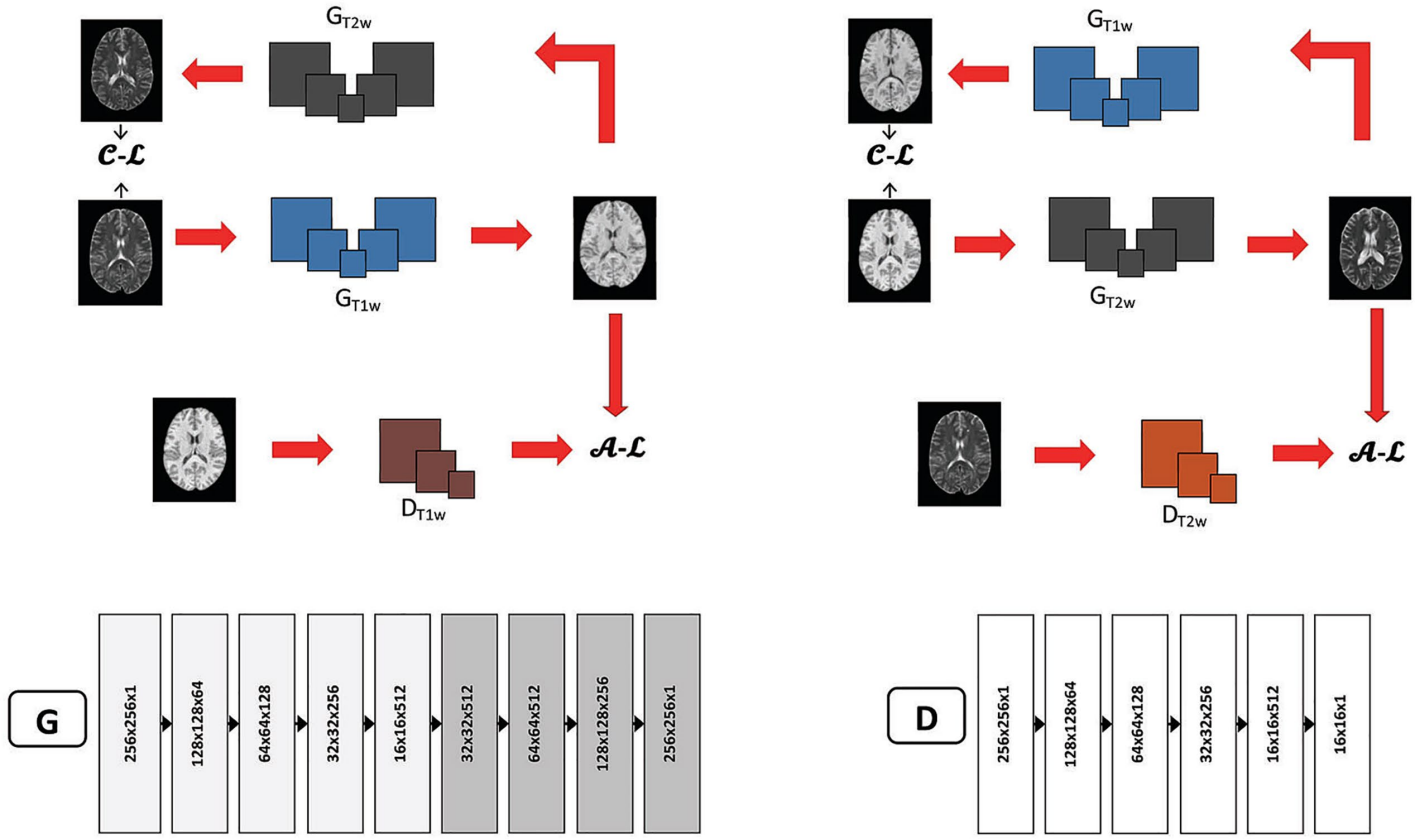
CycleGAN architecture used in the study. The top panels illustrate the procedures for synthesizing T1-weighted (T1w) from T2-weighted (T2w) images (left), and vice versa (right). The bottom panels show the feature numbers corresponding to individual layers of a generator (G) and discriminator (D). C-L, cycle loss between source and reconstructed images; A-L, adversarial loss for a discriminator; G_T2w_ and G_T1w_, generators for T2w and T1w, respectively; D_T1w_ and D_T2w_, discriminators for T1w and T2w, respectively.

We started from a recognized two-dimensional (2D) CycleGAN architecture (Figure 2) (Zhu et al., 2017). Refinement was performed on hyperparameters that often largely impact model stability and output, including the number of down-/up-sampling blocks and the number of residual blocks in the generator, the down-sampling blocks in the discriminator, as well as the initial learning rate and the decay schedule. Candidate settings were chosen based on measures of validation image quality by peak signal-to-noise-ratio (PSNR) and structural similarity index measure (SSIM) across training epochs (Supplementary Figure 1). Accordingly, each model was trained for 200 epochs, where the metrics plateaued, with a batch size of 1 as commonly done in CycleGAN. The initial learning rate was set at 0.0002, which was reduced by a factor of 10 at the 100th epoch. The Adam Optimizer (Kingma and Ba, 2014) was used to minimize loss functions associated with the generators and discriminators. Model training started with individual images at a dimension of 256 × 256 × 1, which was encoded to 16 × 16 × 512 and then gradually decreased to the initial dimension with the generators. The discriminators started with this dimension and decreased to 16 × 16 × 1 eventually. These settings were used in the analysis of all three datasets investigated in this study to ensure consistency. For each study cohort, the prepared data were randomly split at 0.8/0.1/0.1 for training, validation, and testing, respectively, with splitting randomness controlled by a random seed of 42. All training procedures were conducted using an Nvidia A100 80 GB GPU unit with the TensorFlow backend (Abadi et al., 2016).

#### 2.4.2 Model development for CycleGAN with spectral normalization

These models were developed using the same procedures as vanilla CycleGAN, except for the addition of spectral normalization. The latter was added to the convolutional layers of the discriminators by scaling the weight matrices by their maximum singular values (the spectral norms) (Jenkinson et al., 2012). Weight matrices normalized this way were expected to control disproportionate feature amplifications for consistent backpropagation of gradients in the discriminators. To further understand the impact of spectral normalization, imaging contrast variations with or without its addition were also explored based on the healthy HCP data.

### 2.5 Image synthesis with Pix2Pix

The Pix2Pix model was implemented based on a published article (Isola et al., 2016). In contrast to CycleGAN, this method was composed of one pair of generator and discriminator, with the hyperparameters decided based on established practices for GANs and empirical tuning. Specifically, the model was trained for 200 epochs, with a batch size of 1, and hinge loss optimized by Adam. The initial learning rate was 0.0001. Data splits for training, validation, and testing were conducted in the same way as for CycleGAN with each dataset.

### 2.6 Model evaluation

Based on the held-out testing datasets, CycleGAN models with or without spectral normalization were evaluated using three common metrics: PSNR, SSIM, and mean absolute error (MAE). These metrics were calculated per image slice and averaged per person per dataset for subsequent analyses.

### 2.7 Utility evaluation of the synthesized images

Based on the testing T1w brain MRI of MS, utility exploration involved three tasks. The first was lesion detection. Using the TrUE-Net method in FSL (Strain et al., 2024), MS lesions were segmented using T1w and the corresponding FLAIR images, where lesion probabilities below 0.3 were excluded. Segmentation accuracy was assessed using the Dice coefficient and 95th-percentile Hausdorff distance (HD95) for voxel-level overlap of lesions. Lesion count accuracy was measured using the lesion-wise F1 score and the lesion count agreement score. Furthermore, lesion size distribution and detection failure modes were presented for additional understanding of image synthesis methods. The latter included both the failure lesion number and the lesion count variant ratio between synthesized and source images.

The second task was brain volume measurement using T1w MRI following lesion filling as a standard practice, done using the SynthSeg method from FreeSurfer 8.0 (Harvard Medical School, USA). This assessment considered the following four measures: total intracranial volume (ICV), volumes of cortical gray matter, white matter, and cerebrospinal fluid (CSF).

The third was texture analysis around MS lesions using an optimized statistical approach, gray level co-occurrence matrix (GLCM) (Hosseinpour et al., 2022). Using its Scikit implementation in Python, two tested parameters were computed: texture contrast and texture dissimilarity. Both metrics detected subtle structural changes in MS using histology-informed brain MRI (Hosseinpour et al., 2022). Lesion texture was then averaged per subject.

### 2.8 Statistical analysis

Comparing results across imaging sources was performed using one-way ANOVA when normal distributions were satisfied, followed by Tukey correction for multiple comparisons. Friedman’s test was used otherwise. Statistical analyses used the GraphPad Prism package (version 10.4.1, Boston, Massachusetts, USA) and the SciPy package in Python, with *p* ≤ 0.05 set as significance. Comparison between CycleGAN modeling results across datasets was not performed, given their inherent differences.

## 3 Results

### 3.1 Comparable outcomes between synthesized and source images

**Figure 3:**
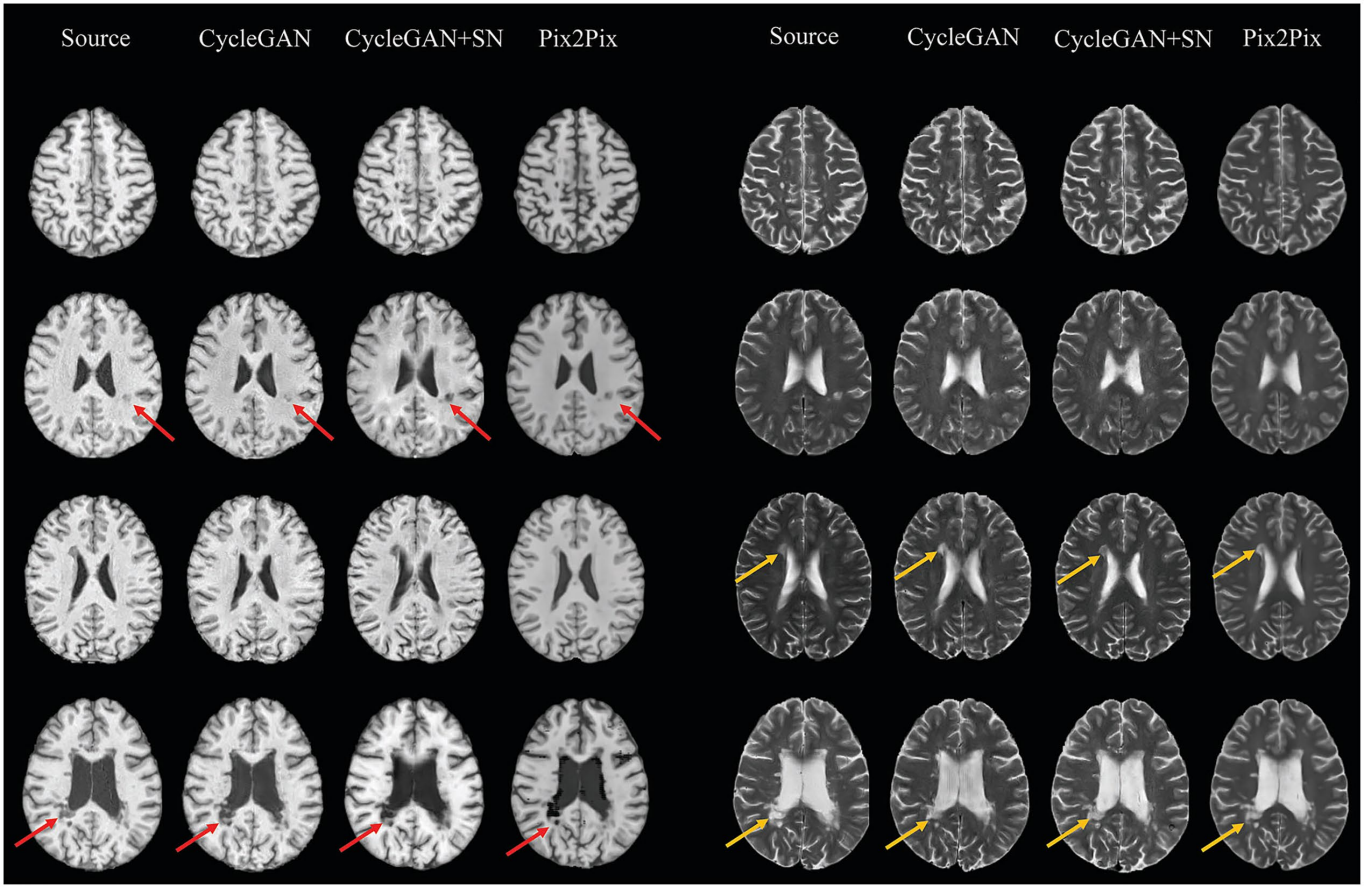
Representative ground truth and synthesized brain MRI of MS based on different methods. CycleGAN models with or without spectral normalization (SN) both replicated lesion areas well in T1-weighted (hypointensity, red arrows) and T2-weighted (hyperintensity, yellow arrows) images, similar to ground truth. The Pix2Pix-generated images show similar findings with slightly higher contrast than the source in a few lesion areas.

Qualitatively (Figure 3), anatomical integrity was well preserved in generated images by both CycleGAN-based and Pix2Pix models, including ventricular morphology and contrast. Lesion size distribution was largely similar based on synthesized images from all models to source images, with a minor exception for relatively small lesions (Supplementary Figure 2). Spectral normalization in CycleGAN appeared to decrease imaging sharpness based on experiments with the testing cohort of HCP, as indicated by lower Laplacian energy and higher Gradient MAE (Supplementary Table 1). Quantitative results supported the overall fidelity of synthesized image from each model, with Pix2Pix performing slightly better than CycleGAN models in certain outputs.

#### 3.1.1 HCP dataset

**Figure 4:**
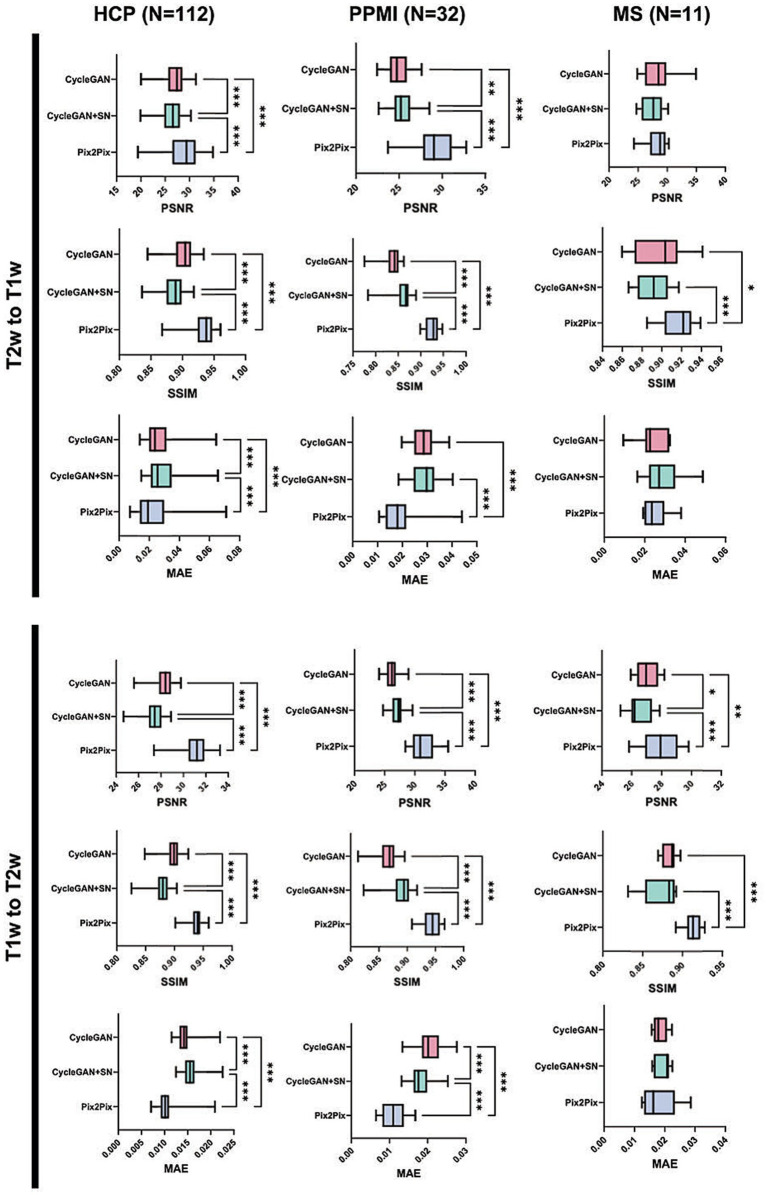
Comparison of CycleGAN models and Pix2Pix by evaluation metric and image synthesis direction. Error bars represent standard deviation. The stars represent significance values: ^*^p ≤ 0.05, ^**^p ≤ 0.01, ^***^p ≤ 0.001. PSNR, peak signal-to-noise ratio; SSIM, structural similarity index measure; MAE, mean absolute error; SN, spectral normalization.

In synthesizing either T1w or T2w images, CycleGAN with or without spectral normalization achieved high PSNR and SSIM, and low MAE, which improved in Pix2Pix, though the effect sizes varied (Tables 1, 2; Figure 4). Specifically, the average values of CycleGAN images ranged from 26.190 to 28.290 versus 28.520 and 31.100 of Pix2Pix for PSNR, 0.879–0.901 versus 0.933 and 0.938 for SSIM, and 0.014–0.029 versus 0.010 and 0.024 for MAE. In addition, CycleGAN without spectral normalization performed better than within both image synthesis directions, with PSNR and SSIM increased by 2.64–3.19% and 1.63–2.09%, and MAE decreased by 4.76–8.30% (all *p* ≤ 0.001).

**Table 1:** Mean (95% confidence interval) testing metrics for the CycleGAN-based and Pix2Pix models on reciprocal synthesis between T1- and T2-weighted brain MRIs across datasets.

Dataset	Translation	Metric	CycleGAN	CycleGAN + SN	Pix2Pix
HCP (*N* = 112)	T2w to T1w	PSNR	26.880 [26.40, 27.37]	26.190 [25.75, 26,63]	28.520 [27.85, 29.19]
		SSIM	0.901 [0.897, 0.904]	0.886 [0.883, 0.890]	0.933 [0.930, 0.937]
		MAE	0.028 [0.025, 0.030]	0.029 [0.027, 0.031]	0.024 [0.021, 0.026]
	T1w to T2w	PSNR	28.290 [28.13, 28.44]	27.410 [27.26, 27.56]	31.100 [30.19, 31.29]
		SSIM	0.898 [0.895, 0.900]	0.879 [0.877, 0.882]	0.938 [0.936, 0.940]
		MAE	0.014 [0.014, 0.015]	0.016 [0.015, 0.016]	0.010 [0.010, 0.011]
PPMI (*N* = 32)	T2w to T1w	PSNR	24.860 [24.37, 25.35]	25.330 [24.80, 25.85]	29.220 [28.41, 30.30]
		SSIM	0.838 [0.832, 0.845]	0.860 [0.852, 0.868]	0.924 [0.919, 0.930]
		MAE	0.028 [0.027, 0.030]	0.029 [0.027, 0.031]	0.019 [0.016, 0.021]
	T1w to T2w	PSNR	26.190 [25.77, 26.61]	27.130 [26.71, 27.54]	31.280 [30.58, 31.98]
		SSIM	0.866 [0.860, 0.873]	0.888 [0.881, 0.896]	0.943 [0.937, 0.948]
		MAE	0.020 [0.019, 0.022]	0.018 [0.017, 0.019]	0.011 [0.010, 0.012]
MS (*N* = 11)	T2w to T1w	PSNR	28.570 [26.69, 30.46]	27.230 [26.00, 28.46]	28.260 [26.99, 29.53]
		SSIM	0.899 [0.882, 0.915]	0.892 [0.881, 0.903]	0.917 [0.904, 0.929]
		MAE	0.025 [0.020, 0.030]	0.029 [0.023, 0.030]	0.025 [0.021, 0.029]
	T1w to T2w	PSNR	26.99 [26.49, 27.49]	26.510 [25.92, 27.11]	28.010 [27.13, 28.89]
		SSIM	0.884 [0.878, 0.890]	0.873 [0.859, 0.886]	0.914 [0.906, 0.921]
		MAE	0.019 [0.017, 0.020]	0.019 [0.018, 0.021]	0.017 [0.014, 0.022]

SN, spectral normalization; PSNR, peak signal-to-noise ratio; SSIM, structural similarity index measure; MAE, mean absolute error.

**Table 2:** Comparison of testing metrics between the two CycleGAN and Pix2Pix models in reciprocal synthesis between T1- and T2-weighted brain MRI across datasets.

Dataset	Modality	Metric	*F*	*p*_ANOVA_	Cohen’s *d* (GAN-SN)	Cohen’s *d* (SN-Pix2Pix)	Cohen’s *d* (GAN-Pix2Pix)
HCP	T1w	PSNR	19.36	<0.001	0.79	−1.32	−0.92
		SSIM	187.42	<0.001	2.37	−4.2	−3.3
		MAE	6.30	0.0021	−0.35	0.84	0.6
	T2w	PSNR	534.53	<0.001	2.72	5.35	5.13
		SSIM	631.56	<0.001	3.73	6.97	6.62
		MAE	241.31	<0.001	−1.69	−3.83	−3.5
PPMI	T1w	PSNR	61.18	<0.001	−0.63	−1.97	−2.39
		SSIM	181.04	<0.001	−2.75	−2.98	−4.76
		MAE	32.77	<0.001	−0.11	1.65	1.76
	T2w	PSNR	109.67	<0.001	−1.47	2.66	2.88
		SSIM	148.35	<0.001	−2.68	3.36	4.7
		MAE	79.89	<0.001	1.09	−2.7	−2.99
MS	T1w	PSNR	1.10	0.35	0.67	−0.57	0.15
		SSIM	4.48	0.02	0.4	−2.03	−1.09
		MAE	1.11	0.34	−0.51	0.47	−0.04
	T2w	PSNR	6.26	0.0054	1.02	1.77	1.41
		SSIM	24.19	<0.001	0.81	3.15	5.13
		MAE	0.58	0.57	−0.56	−0.41	−0.21
		MAE	79.89	<0.001	1.09	−2.7	−2.99

GAN, CycleGAN; SN, spectral normalization (along with CycleGAN); PSNR, peak signal-to-noise ratio; SSIM, structural similarity index measure; MAE, mean absolute error.

#### 3.1.2 PPMI dataset

All CycleGAN images showed competitively high PSNR and SSIM, and low MAE, which also showed improvement in Pix2Pix with different effect sizes (Tables 1, 2; Figure 4). The average values of CycleGANs ranged from 24.860 to 27.130 versus 29.200 and 31.280 of Pix2Pix in PSNR, 0.838–0.888 versus 0.924 and 0.943 for SSIM, and 0.018–0.029 versus 0.011 and 0.019 for MAE. Between models, CycleGAN with spectral normalization showed increased PSNR by 1.89–3.57% and SSIM by 2.56–3.63%, and decreased MAE by 11.02% compared with vanilla CycleGAN, with all *p* ≤ 0.001 except MAE, where *p* ≤ 0.05.

#### 3.1.3 Local MS dataset

Similarly, both PSNR and SSIM were high, and MAE was low with CycleGAN models, with Pix2Pix improved in select comparisons (Tables 1, 2; Figure 4). The average values of CycleGAN images ranged from 26.510–28.570 versus 28.260 and 28.010 of Pix2Pix for PSNR, 0.873–0.899 versus 0.917 and 0.914 for SSIM, and 0.019–0.029 versus 0.017 and 0.025 for MAE. Adding spectral normalization showed no positive impact on CycleGAN outputs, except for a mild improvement in PSNR at T1w to T2w translation.

### 3.2 Equivalent utility between CycleGAN-synthesized and source T1w MRI in MS

**Figure 5:**
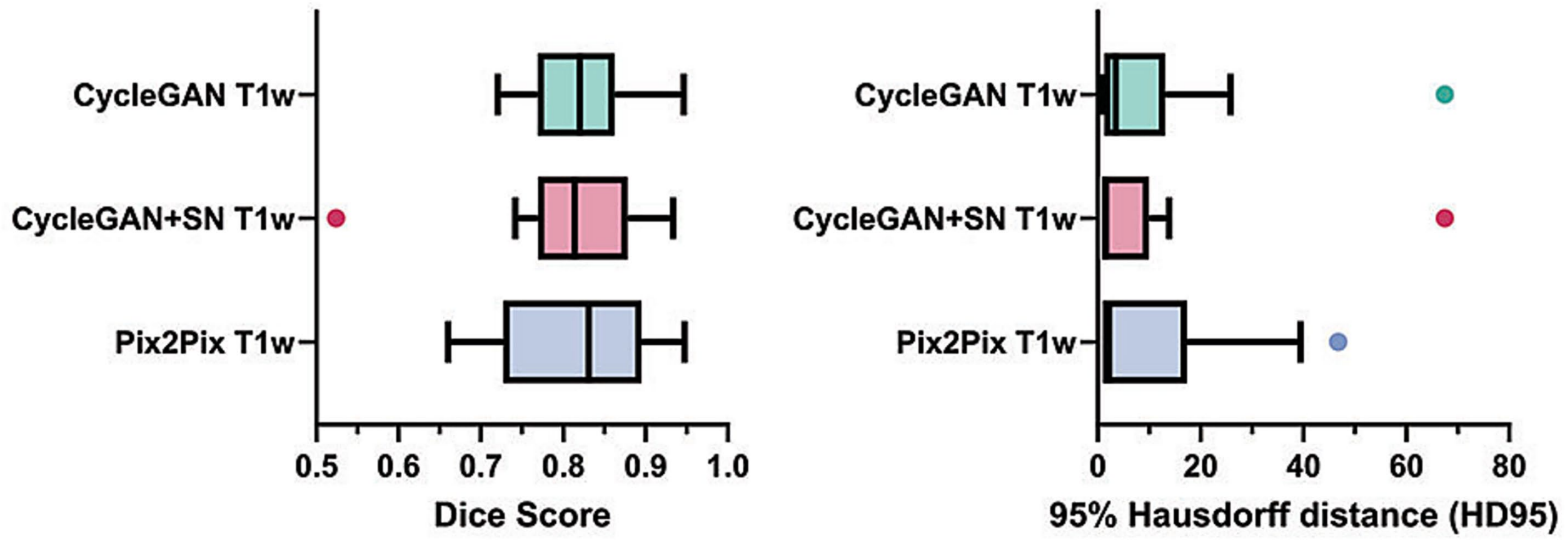
Comparison of lesion detectability between imaging types in multiple sclerosis (MS). The plots show the mean and standard deviation of Dice and HD95 scores. ^*^p < 0.05; ^**^p < 0.01; and SN, spectrum normalization.

In lesion detection, all CycleGAN and Pix2Pix T1w images achieved high Dice and low HD95 scores, with no significant differences between these models (mean Dice > 0.7 and mean HD95 < 10; Figure 5). In lesion count, the lesion-wise F1 ranged 0.638–0.734, and lesion count agreement scores ranged 0.664–0.756 for CycleGAN models and Pix2Pix, respectively (Table 3). The lesion failure rate was low and similar across models, with all lesions detected and the count variant ratio close to or greater than one, indicating high fidelity.

**Table 3:** Lesion count accuracy and failure rates.

Metric	CycleGAN	CycleGAN + SN	Pix2Pix
Lesion-wise F1	0.698	0.638	0.734
Lesion count agreement score	0.664	0.743	0.756
Failure lesion rate (*n*/*N*)	0/11	0/11	0/11
Count variance ratio	1.24	1.65	0.76

*n* refers to the number of lesions failed to detect, and *N* refers to the total number of lesions that exist in the source images.

**Figure 6:**
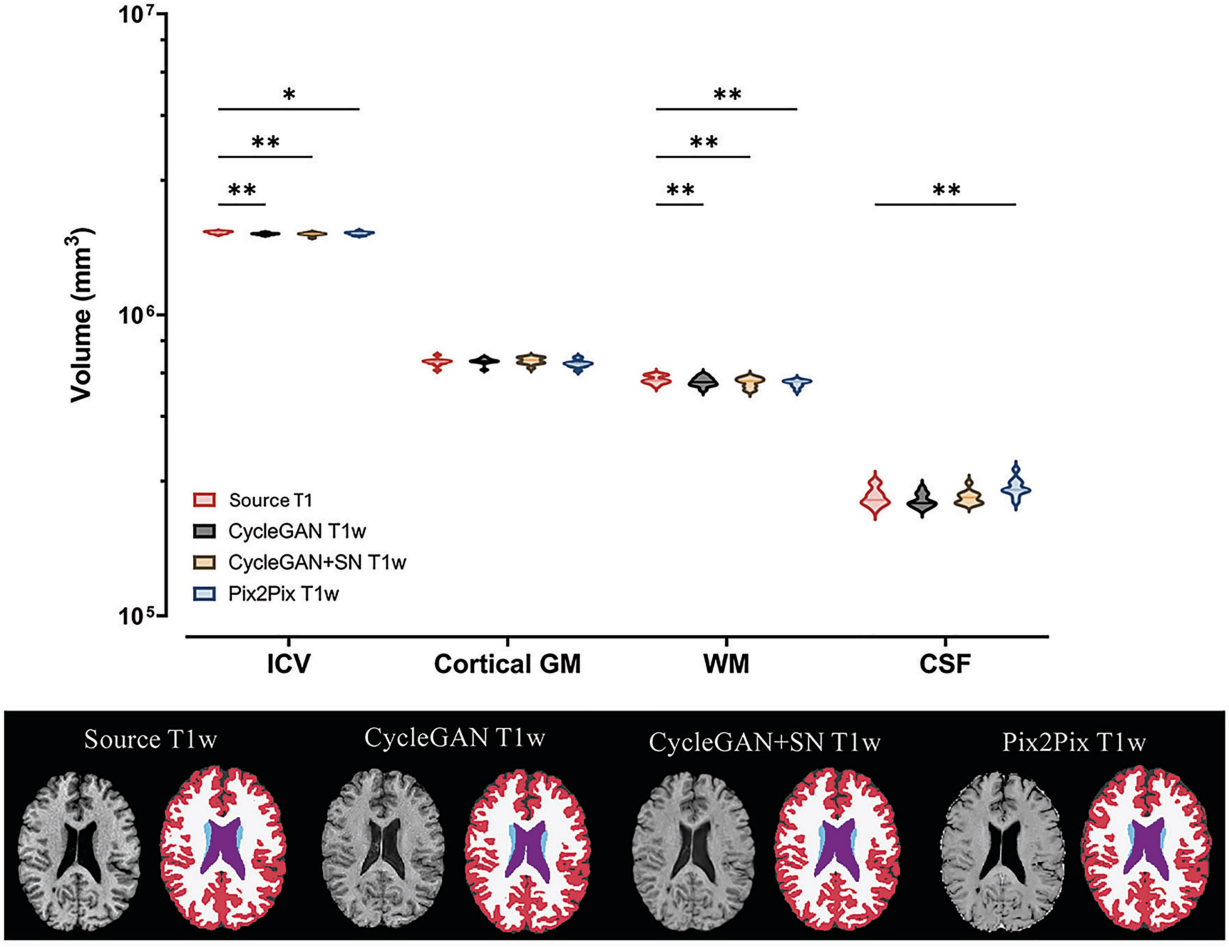
Brain volume measurement by T1-weighted MRI type in multiple sclerosis (MS). The top panel shows log-scaled violin plots of mean and standard deviation intracranial volume (ICV), and cortical gray matter, white matter, and cerebrospinal fluid (CSF) volumes. The bottom panel shows source and synthesized T1-weighted brain MRI, and the corresponding segmentation masks (color maps), from CycleGAN, CycleGAN + spectral normalization (SN), and Pix2Pix. ^*^p < 0.05; ^**^p < 0.01; ^***^p < 0.001; SN, spectrum normalization; and in color maps, red for cortical gray matter, white for matter, and purple for CSF, respectively.

In brain volume measurement, CycleGAN T1w from either version was source-equivalent in cortical gray matter and CSF volumes (*p* > 0.05), but lower than source in ICV and white matter (|*Δ*| ≈ 1.0–1.5%; *p* < 0.01). Pix2Pix T1w volumes showed a similar trend, except for a larger CSF volume than the source (*p* ≤ 0.01; Figure 6).

**Figure 7:**
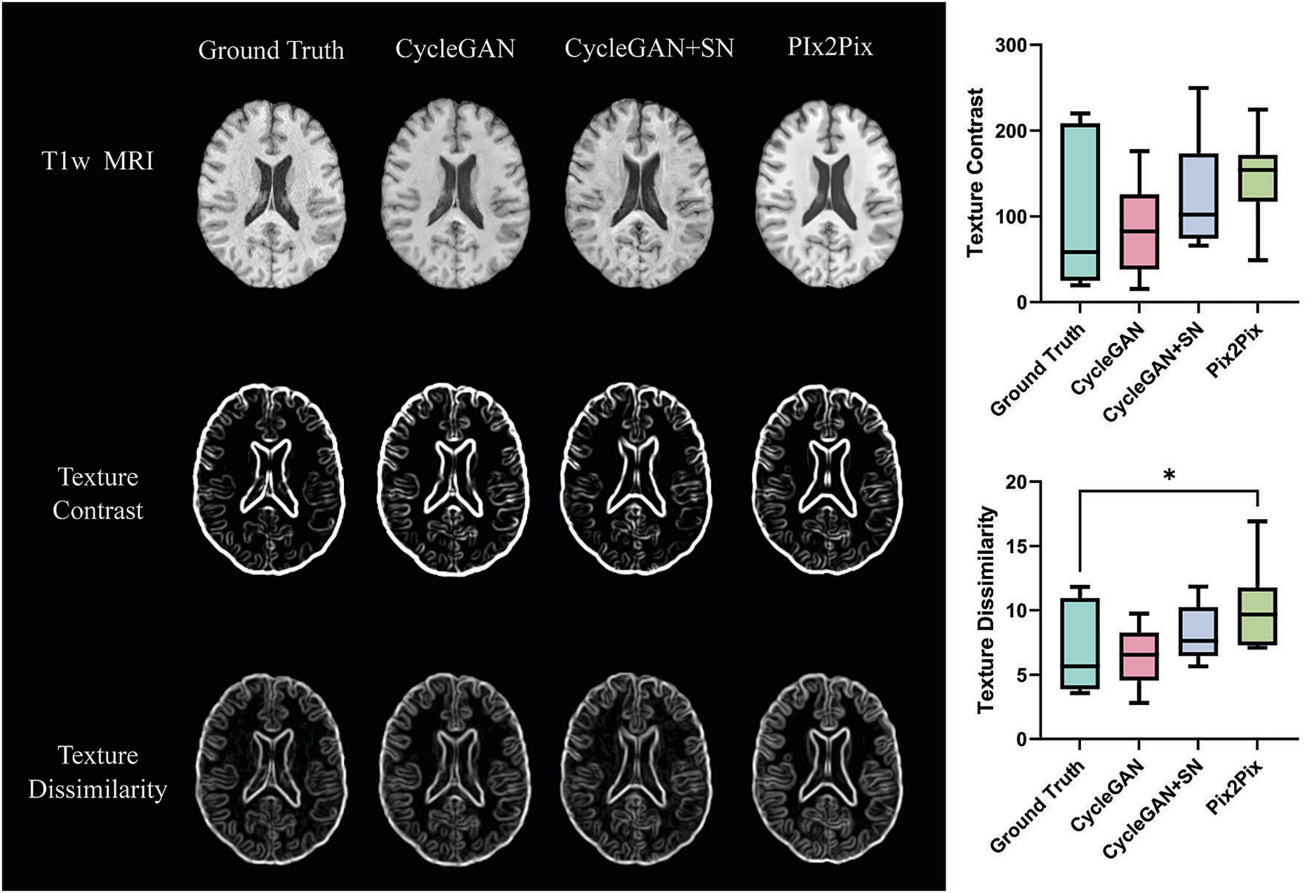
Example images and corresponding texture analysis results by T1-weighted imaging type in multiple sclerosis (MS). Based on the gray level co-occurrence matrix method, the middle and bottom rows represent texture images from the source and synthesized T1 by CycleGAN, CycleGAN with spectral normalization (SN), and Pix2Pix. Box plots on the right show lesion texture computed from the above T1 types from 11 testing MS participants.

In texture analysis, lesion texture contrast was similar between the T1w of all models under investigation compared with source T1w. Lesion texture dissimilarity was higher in Pix2Pix T1w than in source images, while both CycleGAN models showed similar outputs to source results (*p* > 0.05; Figure 7).

## 4 Discussion

Based on investigations using both streamlined and heterogeneous datasets, this study demonstrated the consistency of conditional GAN models in synthesizing T1w and T2w brain MRI. While pending confirmation, the vanilla CycleGAN appeared to be sufficient in synthesizing clinically standard images of MS, and Pix2Pix was mostly superior to CycleGANs in synthesizing images based on streamlined datasets. Utility tests supported the capacity of synthesized T1w images compared with source MRI for structural characterizations in MS.

Deep learning methods have emerged as promising candidates to address image synthesis questions in medical imaging, especially conditional GANs. The capability of CycleGAN was largely attributed to its cycle-consistent loss being minimized, allowing effective learning between the reversible imaging domains under study. Other deep learning methods, such as autoencoders, have also shown potential to synthesize medical images, but such relatively simple networks were mainly used as components of advanced models, including GANs (Wang et al., 2021). Pix2Pix facilitated paired supervision that could directly optimize correspondence between input and target images. Prior work also showed the promise of CycleGAN in advancing brain MRI in MS (La Rosa et al., 2021). However, much of the effort was based on streamlined datasets acquired with well-controlled protocols.

To compare, this study began with two high-quality, consistent datasets. With the healthy HCP data, all of our CycleGAN models achieved competitive results (PSNR > 26; SSIM > 0.8). These values appeared higher than the literature norm, where the PSNR was 22.9–23.2, and the SSIM was 0.78 in T1w to T2w, and 23.9–24.2 and 0.79, respectively, in T2w to T1w synthesis (Bui et al., 2020; Welander et al., 2018). Experiments using the PPMI dataset yielded equivalent results, but they provided an opportunity to further evaluate the potential of the models in handling pathological images based on a highly regarded dataset. Our findings with PPMI were also similar to the literature (PSNR = 19–30; SSIM = 0.65–0.91) (Zhang et al., 2022; Yang et al., 2020; Li et al., 2019). Comparably, the Pix2Pix metrics were mostly greater than those of CycleGANs in our experiments. This was not unexpected because of the benefit of paired supervision used in Pixe2Pix. However, paired training may not always be possible in a clinical setting due to the common phenomenon of missing data and heterogeneity of clinical scans.

In real-world applications, routine scans are more representative than streamlined ones. However, the former also present additional challenges due to inconsistency, complicating *post hoc* quantitative analyses. Image preprocessing may help address general quality-assurance issues but is typically not ideal for handling unavailable data. In the present study, although based on a relatively small sample size, our findings support the potential of vanilla CycleGAN models for generating potentially unavailable brain MRI scans, especially T1w in MS. The associated PSNR (>28) and SSIM (~0.9) were similar to or greater than values from the streamlined datasets and the literature. Pix2Pix could also help when paired images are available.

The results of this study with CycleGAN also suggested the dependence of spectral normalization on datasets. In HCP, including spectral normalization showed a decrease in model performance. In contrast, the same process in PPMI seemed to increase performance, which, however, was not replicated in the MS data. These findings could be due to several reasons, including differences in data characteristics, sample size, and imaging contrast variations. Our pilot experiments with the HCP test data suggested that including spectral normalization in CycleGAN decreased imaging sharpness or contrast. As the HCP scans were from healthy subjects that were already relatively ‘uniform’, this added-smoothing action from spectral normalization might have contributed to worse performance than vanilla CycleGAN. On the other hand, the pathologic PPMI scans would feature increased diversity in imaging contrast and therefore likely benefited from the over-smoothing process. Accordingly, while speculatory, we hypothesized that images with greater heterogeneity would benefit more from spectral normalization in translations with CycleGAN. The same theory did not repeat in our clinical MS dataset, which could be due to the small sample size, requiring further confirmation. In line with our findings, prior studies also showed that spectral normalization might increase (Xu et al., 2022; Xu et al., 2020) or decrease (Zhao et al., 2021) CycleGAN capacity.

Our additional utility experiments in MS also supported the robustness of synthesized images by each of the conditional GAN models investigated in this study. In lesion detection, all models yielded high Dice and low HD95, agreeing with the literature (Rokuss et al., 2025). Likewise, lesion-specific metrics including number count and lesion size distribution appeared to be highly similar to source results, with no considerable differences between models. These findings suggest that a missing T1w sequence could be replaced by synthesized images from these models with little penalty to lesion burden or shape measures.

Brain volume measures also favored the synthesized T1w images. They reproduced source tissue volumes with about 1% deviation in assessing cortical gray matter and CSF, the important metrics in characterizing neurodegeneration as a critical pathology of MS (Rocca et al., 2021). Notably, the Pix2Pix images yielded a higher CSF volume than the CycleGAN models and the source data. This could be due to the hinge loss function used in this model, which made extra emphasis on edge detection.

To understand the utility of synthesized images in characterizing subtle structural patterns, texture analysis was also performed. The lack of difference in lesion texture between CycleGAN-based and source T1w images would further suggest the fidelity of these methods, with vanilla CycleGAN-based values seemingly the closest to source results. The higher lesion texture dissimilarity from Pix2Pix T1w likely reflected greater image heterogeneity than other imaging types, although further confirmation is required. Collectively, although there is a lack of literature for direct comparisons, the concordant results from lesion, volume, and texture analyses support the robustness of our synthesized MS images across scales. The T1w MRI created this way could also serve as a reference in many image preprocessing tasks including co-registration (Smith et al., 2004). By itself, T1w MRI had also been a useful channel in different deep learning applications including disease classification (Zhang et al., 2021).

The study has some limitations. The sample size of our MS dataset was relatively small, limiting generalizability. However, as dramatically different acquisition protocols were involved, the dataset served as a good representation of real-world data. In future studies, our estimation based on a moderate effect size indicated that obtaining 80% power with 0.05 type I error would require 34 subjects per group in a paired design. Additionally, this study focused only on conditional GANs as example deep learning methods, with comparisons only to Pix2Pix. While this might have limited the scope of interpretation, these methods were well-recognized in synthesizing MR images, and our investigation using different types of datasets seemed to support their capacity. Further, our utility evaluation studies were rather exploratory. Nonetheless, our consistent results based on an important yet potentially missing T1w sequence should have provided proof-of-concept evidence. In the future, we plan to confirm the present findings using larger datasets, investigate new methods such as other GAN variants or diffusion models (Koetzier et al., 2024), and assess the role of synthesized images in different applications, including deep learning.

## 5 Conclusion

Our overall findings suggest the consistency and potential of conditional GAN models in synthesizing brain MRI, such as T1w and T2w sequences, across datasets of both low and high heterogeneity. Spectral normalization appeared not mandatory in most of the experiments, including those for synthesizing real-world clinical MS images, deserving further confirmation. Furthermore, CycleGAN-synthesized images appeared feasible for both gross and fine pathological analysis, as demonstrated with T1w brain MRI in MS, supporting further validation.

## Data Availability

The original contributions presented in the study are included in the article/Supplementary material, further inquiries can be directed to the corresponding author.
