# Supplementary material for: CycleGAN models show consistent brain MRI synthesis across datasets supporting downstream tissue characterization in multiple sclerosis
Source: Front Neuroinform. 2026 Mar 12;20:1762794. doi: 10.3389/fninf.2026.1762794 (PMC13018141; doi:10.3389/fninf.2026.1762794)
Supplement: Supplementary file 1 [file Data_Sheet_1.docx]

**Supplementary Table 1.** Image Sharpness experiments with the test set of healthy Human Connectome Project dataset (N = 112).

| Synthesis | Metric | CycleGAN (mean ± sd) | CycleGAN+SN (mean ± sd) | SN worsened (n/N) | Wilcon p | Cohen’s d |
| --- | --- | --- | --- | --- | --- | --- |
| T1 | Laplacian energy | 0.147 ± 0.037 | 0.127 ± 0.027 | 94 | <0.001 | -0.92 |
|  | Gradient MAE | 1.650 ± 0.159 | 1.750 ± 0.154 | 82 | <0.001 | +0.68 |
| T2 | Laplacian energy | 0.137 ± 0.013 | 0.129 ± 0.015 | 99 | <0.001 | -1.30 |
|  | Gradient MAE | 1.008 ± 0.092 | 1.104 ± 0.092 | 110 | <0.001 | +2.57 |

Note: sd: standard deviation; n: number of subjects worsened by adding spectral normalization (SN) to the CycleGAN model; Wilco: Wilcoxon test.


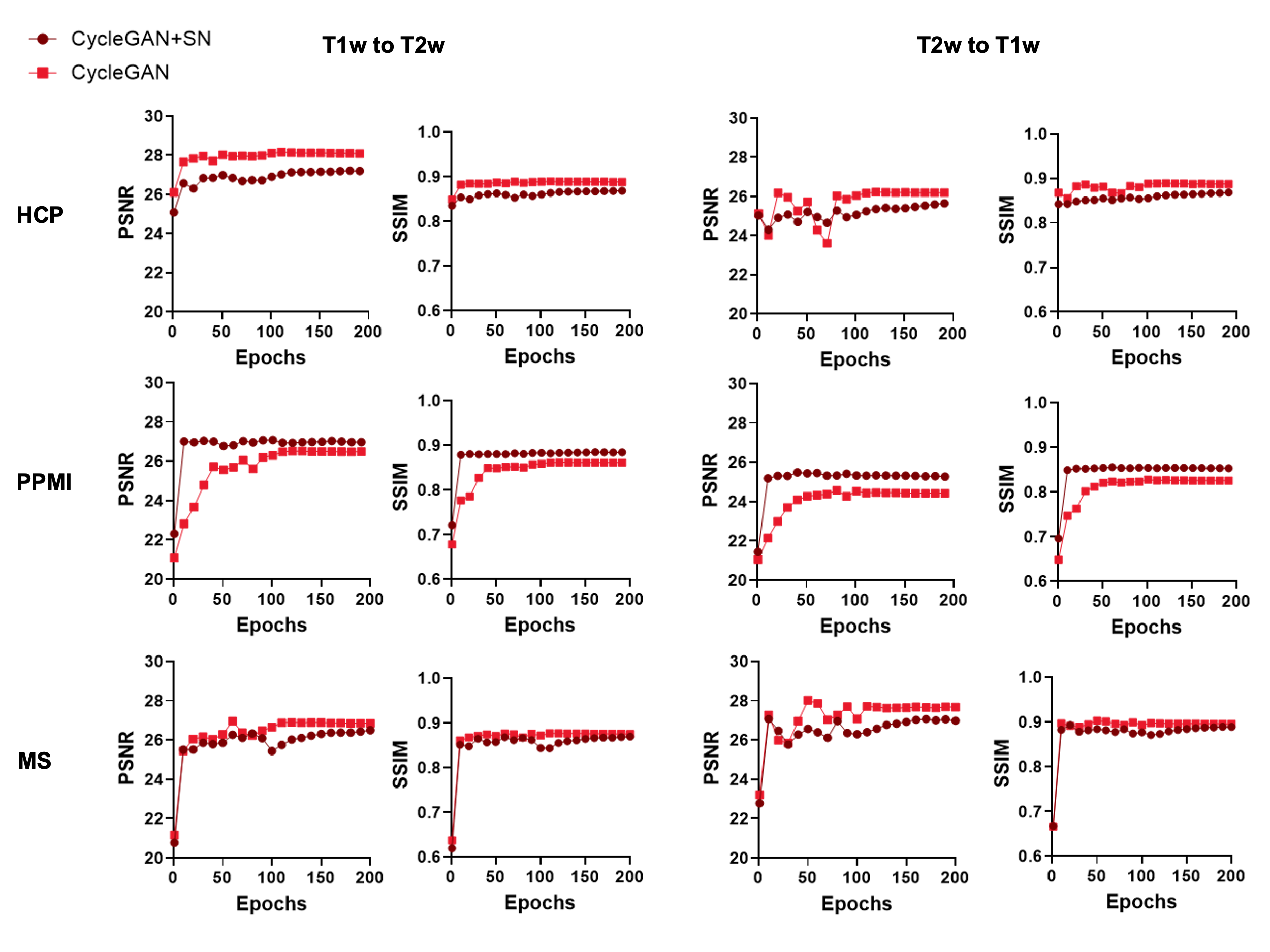


**Supplementary Figure 1** Validation outputs by epoch for both image synthesis directions per dataset. In each plot, the curves are shown for CycleGAN, both with and without spectral normalization (SN). Note: PSNR: peak signal-to-noise ration; SSIM: structural similarity index measure; and MAE: mean absolute error.


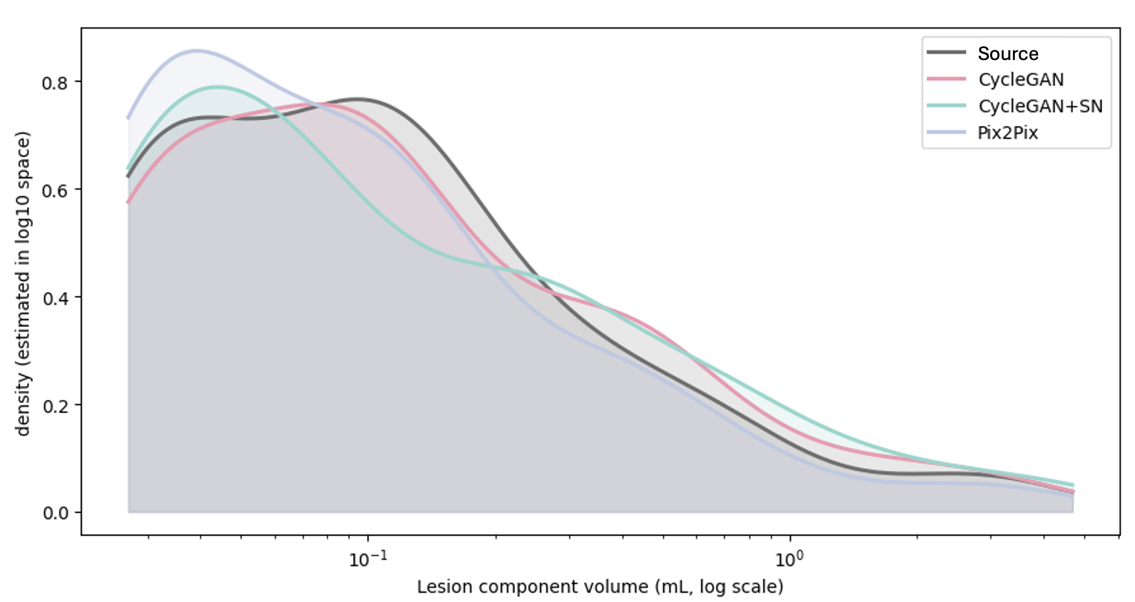


**Supplementary Figure 2**: Lesion size distribution from the test dataset of multiple sclerosis using synthesized or source T1-weighted brain MRI (N=11). Note: SN refers to spectral normalization.
